# Safeguarding patients from technology-facilitated abuse in clinical settings: A narrative review

**DOI:** 10.1371/journal.pdig.0000089

**Published:** 2023-01-04

**Authors:** Isabel Straw, Leonie Tanczer

**Affiliations:** 1 Institute of Health Informatics, University College London, London, United Kingdom; 2 Gender and IoT, UCL Department of Science, Technology, Engineering and Public Policy (UCL STEaPP), London, United Kingdom; Tsinghua University, CHINA

## Abstract

Safeguarding vulnerable patients is a key responsibility of healthcare professionals. Yet, existing clinical and patient management protocols are outdated as they do not address the emerging threats of technology-facilitated abuse. The latter describes the misuse of digital systems such as smartphones or other Internet-connected devices to monitor, control and intimidate individuals. The lack of attention given to how technology-facilitated abuse may affect patients in their lives, can result in clinicians failing to protect vulnerable patients and may affect their care in several unexpected ways. We attempt to address this gap by evaluating the literature that is available to healthcare practitioners working with patients impacted by digitally enabled forms of harm. A literature search was carried out between September 2021 and January 2022, in which three academic databases were probed using strings of relevant search terms, returning a total of 59 articles for full text review. The articles were appraised according to three criteria: (a) the focus on technology-facilitated abuse; (b) the relevance to clinical settings; and (c) the role of healthcare practitioners in safeguarding. Of the 59 articles, 17 articles met at least one criterion and only one article met all three criteria. We drew additional information from the grey literature to identify areas for improvement in medical settings and at-risk patient groups. Technology-facilitated abuse concerns healthcare professionals from the point of consultation to the point of discharge, as a result clinicians need to be equipped with the tools to identify and address these harms at any stage of the patient’s journey. In this article, we offer recommendations for further research within different medical subspecialities and highlight areas requiring policy development in clinical environments.

## Author summary

Technology-facilitated abuse describes the misuse of digital systems such as smartphones or other Internet-connected devices to harm individuals. The proliferation of these devices within our environment, exacerbated by the COVID19 pandemic, has increased the risks of technology-facilitated-abuse for vulnerable members of society. These forms of abuse are on the rise, with perpetrators using digital technologies such as GPS Tags, and device spyware tools to monitor and control individuals. Vulnerable individuals frequently perceive medical settings as a place of safety and thus healthcare professionals have a role in providing both medical and psychosocial care to ensure their wellbeing. At present, existing clinical and patient management protocols are outdated and do not address the emerging threats of technology-facilitated abuse. Throughout our examination of the existing literature we explore the guidance that is available to healthcare practitioners who are caring for affected populations and make concrete recommendations that are urgently needed to effectively safeguard vulnerable patient groups.

## 1.0 Background

Vulnerable patients frequently perceive medical settings as a place of safety. Clinicians, thus, have a role in providing both medical and psychosocial care to ensure their wellbeing. Clinical safeguarding protocols offer essential guidance to practitioners navigating high risk scenarios. These guidelines require regular updates to respond to evolving changes in society. For example, in recent years, pediatric safeguarding guidelines have been amended in response to increasing rates of knife crime, gang violence and drug trafficking in the UK [1–3]. Williams described the growing threat of County lines and drug trafficking to young people and advocated for the improved education of healthcare staff interacting with these groups [4]. While technology-facilitated abuse has evolved at a parallel rate to these threats, it has not received the same level of attention in the medical setting.

Technology-facilitated forms of abuse are on the rise, with perpetrators adapting digital technologies such as smartphones and drones, trackers such as AirTags and spyware tools such as parental control software to cause harm [5]. The impact of technology-facilitated abuse on patients may not always be immediately obvious to clinicians. For instance, smart, Internet-connected devices (aka ‘Internet of Things’ or ‘IoT’) have been showcased to be misused in domestic abuse cases to inflict physical harm [6]. Examples of this IoT-facilitated abuse (Table 1) include the manipulation of smart thermostats and air conditioning systems to expose victims to extreme temperatures, or cause distress through smart systems ability to be remotely controlled [7–9]. Furthermore, the anti-privacy nature of some smart IoT devices can be manipulated for the purpose of occupancy detection. The exploitation of home devices, such as off-the-shelf smart electricity meters, to track individuals within their homes, increases the vulnerability of victims of harassment and stalking [10,11].

**Table 1:** Common terms used in the field of technology-facilitated abuse.

**IoT-facilitated abuse**	Smart surveillance and IoT-facilitated abuse include the use of “connected” devices that communicate through a network to monitor people or places. Such devices may include thermostats, security cameras, motion detectors, smart locks, GPS trackers and children’s toys. An abuser could misuse connected devices to monitor, harass, isolate, and otherwise harm a victim [6,17].
**Cyberstalking**	Cyberstalking includes behaviors of surveillance, monitoring, repeated contact, and impersonation [17]. Technology can act as a facilitating force in stalking instances.
**Cyberbullying**	Cyberbullying is the use of technology to facilitate bullying behavior, which is to deliberately and repeatedly engage in hostile behavior to hurt a victim socially, psychologically, or even physically [18].
**Doxing/Doxxing**	Publicly searching and consequently publishing private information that can be used to identify or intimidate someone [18].
**Sextortion**	Sextortion, or sexual extortion, is a form of blackmail where a perpetrator threatens to reveal intimate images of their victim unless the affected party gives in to their demands [18].
**Sexting**	Sexting is a term used to describe the voluntary act of sending and receiving sexually explicit text messages, photographs or videos, mainly through a mobile device or via platforms such as social media outlets [17–18].
**Non-consensual image-sharing/ “Revenge pornography”**	Non-consensual image sharing, image-based sexual abuse, or “revenge pornography” refers to the sharing or distribution of sexual, intimate, nude, or semi-nude photographs or videos without a person’s permission [17–18].
**Deepfake**	A deepfake is an extremely realistic—though fake—image or video that shows a real person doing or saying something that they did not actually do or say [18].
**Trolling**	Trolling is the process of indiscriminate targeting, involving any subject matter [12].
**Spoofing**	Spoofing is a term that describes masking or hiding one’s actual phone number so that another phone number (chosen by the user) shows up on the recipient’s caller ID [17].
**Impersonation**	Abusers may create accounts in a victim’s name or manipulate technology in a way that makes it seem like a communication is coming from the victim or another actor they pretend to be.
**GPS Monitoring**	A Global Positioning System (GPS) is a network of satellites that provides location information to many common devices such as smartphones or car navigation systems. Different digital products–including dedicated tracking systems—can include GPS technology, enabling abusers to place the device, for instance, into someone’s purse and misuse the technology to track a victim’s location [12,17–18].

Clinical syndromes that arise from the intersection of technology and human physiology is a relatively new area in the medical domain. For example, Ronen and Shamir describe IoT device hacks that can result in illness [9]. The authors demonstrate that the tampering smart lights at specific frequency ranges can be used to induce seizures in people suffering from photosensitive epilepsy [8]. The risks of technology-facilitated abuse can consequently be severe, with online harassment having been linked to increased rates of victim homicide and suicide [7,12–13]. Furthermore, the use of electronic surveillance and Global Positioning System-tools (GPS; e.g., in vehicles and baby monitors) has been shown to compromise victim’s safety when accessing support services [7,14–15].

Technology-facilitated abuse is increasing in prevalence, with Refuge, the UK’s largest domestic violence charity, stating that 72% of service users experience abuse through technology [16]. At present, clinicians receive little training in digital safeguarding, despite their regular consultations with groups impacted by these harms. The lack of awareness and inadequate safeguarding guidance is limiting the care that medical professionals can provide. While clinicians cannot be literate in all technical issues, a basic understanding of how technology-mediated harm may manifest is essential. Additionally, the safeguarding protocols available in medical settings require an update regarding technology-related challenges, so that practitioners can access these resources when necessary.

In this review, we examine the existing literature on technology-facilitated abuse in clinical settings and evaluate the safeguarding guidance that is available to healthcare practitioners working with vulnerable groups. We aim to identify gaps in the existing clinical literature and make recommendations for improving safeguarding practice in the future.

## 2.0 Methodology of literature review

Scopus, Pubmed, and Cochrane library were chosen as the target academic databases due to their use by healthcare specialists. Search queries were formed from relevant terms including “technology”, “safeguarding”, “digital” and “abuse” and used to search these databases, replicating the methodology of similar review studies [19]. S1 Table details the search query terms, number of results, and content details of each returned article. The returned articles were appraised according to three criteria:

1. **Does the article focus on technology-facilitated abuse?**
2. **Does the article look at clinical settings?**
3. **Does the article consider the safeguarding needs of patients against the harms of technology-facilitated abuse?**

S1 Table reports the criteria that each article met, if all three criteria were met the article was included in the results list.

## 3.0 Results

Our searches across all three databases returned 61 results, from which two duplicates were removed, leaving 59 articles for review. Of these 59 articles, 17 articles met at least one of our criteria, while only one article met all three criteria. The one article that met all three criteria (‘*How Public Health Nurses’ Deal with Sexting among Young People*: *A Qualitative Inquiry Using the Critical Incident Technique’*) examined the role of public health nurses (including family nurses, health visitors and school nurses) in addressing adolescent digital health needs. The study highlights the importance of these practitioners in addressing digital harms and the lack of guidance around digital safety that currently exists for these professionals. Of note, the study is limited by its narrow emphasis on sexting as opposed to wider forms of technology-facilitated abuse.

A significant number of the returned papers concentrate on the role of technology in preventing, detecting, or documenting abuse, as opposed to zooming in on the impact of technology-facilitated abuse itself. Additionally, the papers which centered on the harms resulting from digital systems, focused on school settings or services for looked after children, with little attention given to adults and no attention given to medical settings [20]. Several international articles were also returned, illustrating the global scope of these technological challenges. In ‘*Technology-facilitated harm to individuals and society*: *Cases of minor’s self-produced sexual content in Russia’* the author reports the growth of technology-facilitated abuse in Russia and the lack of appropriate safeguards for addressing these harms.

The studies that honed in on clinical settings, did not discuss technology and abuse in the context of safeguarding. For example, ‘*A Discussion of the Use of Virtual Reality for Training Healthcare Practitioners to Recognize Child Protection Issues’* by Drewet et al. (2019) considered the role of virtual reality for training clinicians in hospital safeguarding. Nevertheless, it does not touch upon the abuse that results from digital devices nor the implication they may have on child maltreatment.

UK studies that fixate on safeguarding against technology-facilitated abuse did not consider the medical setting. ‘*Understanding Revenge Pornography*: *A National Survey of Police Officers and Staff in England and Wales’* by Bond et al, highlighted the lack of understanding and need for additional training around technology-facilitated abuse but focused solely on police forces [21]. Furthermore, Hackett et al provide a comprehensive overview of the trends in cyberviolence within society. However, attention is not given to the medical domain [20]. Our results demonstrate that most studies that focus on safeguarding against technology-facilitated abuse arise from different disciplines, and equivalent guidance does not yet exist for healthcare practitioners working in clinical environments.

## 4.0 Discussion

At present, there is a lack of guidance for healthcare practitioners who work with patients affected by technology-facilitated abuse. To address this research gap, we focus the remainder of this paper on drawing information from the grey literature and other specialist domains, to highlight how these resources may be adapted to the medical setting. We discuss the impact of technology-facilitated abuse on both adult and pediatric patient populations in hospital and community settings, in addition to examining patient groups that are at an increased risk of harm. We conclude by providing a series of recommendations for practicing clinicians and for researchers looking to improve evidence-base in this domain.

### 4.1 Domestic abuse, youth violence and technology facilitated abuse

IoT-facilitated abuse includes the use of smart connected devices to monitor and/or harm individuals [6,17]. Smart devices are gadgets connected to one another through the internet, such as smart fridges, home security cameras, and automated lights. COVID-19 catalyzed the proliferation of these technologies, with sales of smart devices increasing 30% on last year [6,13]. Yet, while these tools are advertised for their proposed safety and convenience, they are also providing new avenues for violence and domestic abuse [6,12,14]. Voice controlled assistants, smart light bulbs, and video-capturing doorbells have all been manipulated for the purpose of monitoring and controlling the communication and behavior of abuse victims [6,9,15]. Riley reports the dangers of Internet-connected locks (by restricting movement within the home), the use of smart thermostats to abuse partners (by imposing extremes of temperature) and the harm caused by smart speakers (by blasting loud noise in the night) [6,14].

For the clinician, these cases highlight two important considerations; on the one hand, it is necessary to understand the changing dynamics in which violence and abuse may manifest itself, and on the other hand, we need to reconceptualize our understanding of safe environments when discharging a patient. Firstly, the physical impact of domestic abuse often presents as blunt injury caused by physical assault. Yet, the reported use of smart home thermostats, light installations, and sound systems to harm victims presents new forms of injury (physical and beyond) that are not usually accounted for in abuse assessments. Secondly, when discharging patients, it is necessary to reflect on the safety of their home environment. The integration of potentially harmful digital devices within the home setting needs to be assessed when making discharge decisions. Furthermore, when referring a patient to a place of safety (e.g., a domestic violence shelter), GPS trackers and other forms of surveillance such as smart watches need to be scrutinized to ensure that the patient can be transferred safely.

### 4.2 Clinical assessment and patient risk

Technology-facilitated abuse can have a long-lasting effect on victims, which is particularly relevant to GP and hospital clinicians who work with patients over prolonged periods. Victims experience a range of abuses, from general harassment, to digital surveillance using spyware and tracking devices, and sextortion (having intimate images or videos shared without their consent) [14]. GPS trackers have been a growing phenomenon in domestic violence cases, including reports of trackers being place in children toy’s and prams [14]. The significance of these harms cause victims to undergo serious states of anxiety and trauma, putting individuals at a heightened risk for future psychological symptoms, self-injury, suicidal ideation [12].

In addition to the increased mental health risk that technology-facilitated abuse creates, early research has started exploring the causal pathways between technology-facilitated abuse and homicide [22]. Instances of technology-facilitated abuse are linked to domestic homicide and have been identified as an emerging trend by death review panels of family violence [22–23]. Victims are also less likely to recognize this form of abuse as an indicator of danger, highlighting the importance of safeguarding these patients [23].

Digital risks vary from patient assessment to patient management. Clinicians must also question the hazard that is present at the point of patient consultation. In 2018, one of the first court cases for smart-home facilitated abuse resulted in the prosecution of man who used a tablet microphone to eavesdrop on his partner and then assaulted her [6]. The deployment of smart devices to eavesdrop on victims who are seeking help poses a significant challenge to clinicians working to support these patients.

When evaluating the risk of violence to a patient, we must also consider any vulnerable individuals who may also be at risk through their relationship to the victim. Over one quarter (27%) of domestic violence cases involve technology-facilitated abuse of children [24]. The abuse has negative consequences on children’s mental health, their relationships with the non-abusive parent, their educational attainment, and their daily activities [24].

### 4.3 Impact on pediatric patients

In our current society, individuals are engaging with digital systems constantly and it is obsolete to perceive individuals having separate “online” and “offline” lives. For pediatricians, it is consequently essential to update their practices. Clinicians are urged to improve their digital literacy to connect with–especially younger—patients and understand the challenges they are facing. The EU Kids Online Survey asked children across Europe in 2010–2011 to described what upset them online and found several disturbing trends [25]. Below are a few of the responses [25]:

- ‘A mate showed me once a video about an execution. It was not fun’ (Boy, 15).
- ‘Animal cruelty, adults hitting kids’ (Girl, 9).
- ‘Showing images of physical violence, torture and suicide images’ (Girl, 12).

The more recent EU Kids Online Survey (2020) builds on the previous study and highlights the changing landscape of data misuse as it applies to young people, specifically in the context of GPS surveillance [26]. In response to being asked whether “*Someone found out where I was because they tracked my phone or device*”, children answering yes ranged from 1% (Croatia) to 9% (Malta). In the latest report we also see a focus on excessive internet use and the impact of the internet on young people’s socialization. In answer to *“I have spent less time than I should with either family*, *friends or doing schoolwork because of the time I spent on the internet”*, affirmative responses ranged from 4% (The Slovak Republic), to 21% (Belgium) [26].

The impact of technology-facilitated abuse on children may manifest as emotional distress, anxiety, suicidal ideation [12]. Koubel reports the exacerbation of mental health risks born from websites that encourage self-harm, eating disorders, and suicide [27]. Furthermore, technology-facilitated dating abuse and sextortion is increasing amongst adolescent populations. With 10% of children being affected by sexual solicitation online, the problem is widespread and under investigated [12]. As reported by Stonard et al in “*They’ll Always Find a Way to Get to You”*, digital devices are playing an increasing role in relationship abuse amongst young people [13].

In response to the risk to children, schools have brought in a range of digital safety initiatives which may provide inspiration to safeguarding professionals in clinical environments, including the use of e-safety representatives, information material, and annual talks with members of the police [28]. Lloyd reports the growth of adolescent sexual abuse through digital image sharing in schools, which has given rise to a review of educational policies [29]. As explained by Lloyd, school policies need a digital update to ensure safer school environments; the same is needed in the clinical environment to create digitally safe clinical spaces [29].

### Patient groups at increased risk of digital harm

Certain patient groups are particularly at risk, including hospitalized children, those with intellectual disability, as well as elderly patients, and religious groups. Sawyer et al. report the benefits that technology brings to children who are hospitalized for long periods of time by providing socialization and connection during these periods of isolation [30]. Yet the increased exposure to technology also puts these groups at a heightened peril of digital exploitation, a concern which currently is not being addressed in hospital settings. Despite some hospital restrictions on social messaging sites for pediatric patients, patients (particularly adolescents) reported navigating around these restrictions to access these websites [30]. At present, pediatric patients often have a greater digital literacy than those charged with safeguarding them, a fact that makes hospital safeguarding measures more difficult to measure and evaluate.

In clinical practice we further frequently encounter patients with intellectual disability. These patients often rely on digital technology for social connection and communities of interest. The manipulation of technology can therefore disproportionately affect this group [14]. Victimization of patients with chronic conditions or disabilities is particularly prevalent and the rise in disability hate crimes mediated through technology has a severe negative repercussions on physical and psychological health [31–32]. Alhaboby et al. report the negative health impact resulting from these forms of technology-facilitated abuse including anxiety, psychosomatic illness and self-harm [32].

Additionally, the elderly population faces an increased risk of digital exploitation due to lower rates of digital literacy amongst this patient group. For those working in the community, digital risks may differ to those in hospital settings. As reported by Fisk, the use of surveillance technologies in care homes can both protect and harm older patients by intruding on their privacy [33].

Lastly, specific religious group are at greatest risk of online harm, with increasing rates of online antisemitic and Islamophobic content being reported in the UK [12]. Furthermore, researchers have observed an increase in online hate towards migrants, refugees and asylum seekers [12]. Minority patient groups, including racial and ethnic minorities, LGBTQ+ patients and neurodiverse patients are all at greater risk of abuse in both the physical and digital environment.

## 5.0 Conclusion

The role of the clinician is continuously changing and now also affected by the significant relevance of digital systems. We have discussed the impact of technology on: (a) patient presentation, including the physical injuries from temperature and noise manipulation in smart homes (b) patient consultation, including the challenges of safe assessment in the context of surveillance/tracking; and (c) patient discharge, exploring the way in which we need to reconsider our understanding of risk assessments and safe homes in technological settings. At present, there is little research into the manifestation and risks of technology-facilitated abuse in clinical environments. A greater understanding of the links between digital risk factors and patient outcomes is necessary for clinicians to provide effective and timely patient care.

### Recommendations for practice

We have discussed several examples of technology-facilitated abuse throughout this essay, some of which are relevant across the healthcare environment whereas others may be more apparent in a specific specialty. Across all healthcare settings, practitioners may consider removing electronic devices from the consultation room when engaging in a sensitive consultation–in the absence of clear guidance, this may be a temporary solution to circumventing the risks of device spyware. Further, in any setting where a vulnerable person may be moved to a refuge or safe location, healthcare practitioners must know to screen for the presence of GPS technologies or electronic surveillance. In the absence of healthcare guidelines, practitioners can look to recommendations from domestic violence sector. Domestic violence charity ‘Refuge’ provides comprehensive resources on technology-facilitated abuse which could be integrated into guidelines within the healthcare sector [34]. Refuge’s ‘Home Tech Tool’ identifies exploitable devices in an individual’s residence; the ‘Digital Break Up Tool’ provides security options across a range of online media platforms (e.g., economic, social and fitness apps); and their online resources provide educational material on how to identify harms such as cyberstalking [34].

In General Practice or Emergency Medical settings where practitioners frequently consult victims of abuse or domestic violence, clinicians would benefit from an educational update on the potential presentation of technology related harms. The physical abuse resulting from the manipulation of lighting, heating and sound systems may impose different physiological complaints which may not be elicited through standard medical history-taking. A technology-focused update to the procedures for taking these sensitive histories is especially necessary within these specialties, to ensure the scope of abuse is being captured.

The heightened risks faced by pediatric patients highlights the need for tailored changes within in the child health domain. We suggest that policy makers take initiative from the education sector, where dedicated online safety officers play a role in safeguarding within schools. An equivalent professional with the appropriate expertise situated within the medical setting could offer guidance to practitioners on complex technical scenarios and support vulnerable patients, such as long-stay patients who are at greater risk due to their isolation within the hospital.

A growing body of research within psychiatry is exploring the impact of digital technologies on mental health–these enquires must extend to the topic of technology-facilitated abuse. The long-lasting psychological sequalae of technology-facilitated abuse are unknown and there is little literature describing the most effective support for patients who have experienced mental distress from these harms. To collect this data, psychiatrists will need to integrate a digital history into their patient assessment and further research is required to ascertain the mental health trajectories, and possible interventions, that result from technology-facilitated abuse.

The results of our review indicate that technology-facilitated abuse in clinical settings is an under-researched area and in need of greater attention. In addition to wider academic research, the following recommendations are applicable across the healthcare domain and would improve clinical practice within each sub-specialty this space.

- **Education**: Education initiatives are needed in clinical settings to increase awareness of technology-facilitated abuse. The integration of training modules into medical school and professional development curriculums would help serve this purpose.
- **Cross-Disciplinary Initiatives**: Collaboration projects between the police and schools have been beneficial in improving teachers understanding of digital harms. Similar initiatives are needed in medical settings to update the knowledge of healthcare practitioners.
- **Research**: Research is needed at the intersection of clinical practice and technology-facilitated abuse, to identify how patient risk assessments and safeguarding guidelines may need to be adapted based on the link between digital factors and patient morbidity/mortality.
- **Hospital Policies and Clinical Guidance**: Specific guidance is urgently needed in clinical settings regarding how we can adapt patient consultation, assessment, and management to account for digital risks. Community and hospital protocols need updating in accordance with the best available research on technology-facilitated abuse.
- **Quality Improvement (QI) and Audits**: Healthcare staff are expected to contribute to QI projects as part of their professional development. QI projects that focus on technology-facilitated abuse would be an effective means to evaluate and improve existing practice in local settings.
- **Alignment with Policy**: Globally, dedicated online harms legislations are emerging, including the UK Online Safety Bill. It would be important for clinical professions to get involved in these developments and ensure that these developments are also reflective of the needs and demands of the medical profession.

## Supporting information

S1 Table Academic Databases and Search Query Terms used for Literature Search. (DOCX)

S1 PRISMA Checklist The PRISMA (Preferred Reporting Items for Systematic Reviews and Meta-Analyses) checklist consists of 27 items used for assessing systematic reviews. (PDF)
