# Supplementary material for: Safeguarding patients from technology-facilitated abuse in clinical settings: A narrative review
Source: PLOS Digit Health. 2023 Jan 4;2(1):e0000089. doi: 10.1371/journal.pdig.0000089 (PMC9931347; doi:10.1371/journal.pdig.0000089)
Supplement: S1 Table — (DOCX) [file pdig.0000089.s001.docx]

**S1 Table**

| **Academic Databases and Search Query Terms used for Literature Search** | | | | |
| --- | --- | --- | --- | --- |
| **Academic Service** | **Terms** | **Results** | **Study** | **Criteria met [1,2,3] & Included in final results [y/n]** |
| **PubMed** | “Safeguarding” AND “Technology” AND “Abuse” | **26 results** | | |
|  |  | 1 | Antisocial behavior and conduct disorders in children and young people: recognition and management | [0] [n] |
|  |  | 2 | Improving practice in safeguarding at the interface between hospital services and children’s social care: a mixed-methods case study | [1, 3] [n] |
|  |  | 3 | Exploring staff conceptions of prevention and management practices in encounters with staff-directed aggression in supported housing following education and training | [0] [n] |
|  |  | 4 | Therapeutic and reproductive cloning--implications and recommendations | [0] [n] |
|  |  | 5 | Recommendations for Preventing Child Sexual Abuse in Youth-Serving Organizations: Implications From an Australian Royal Commission Review of the Literature | [0] [n] |
|  |  | 6 | Assessing for domestic violence in sexual health environments: a qualitative study | [0] [n] |
|  |  | 7 | When Should Screening and Surveillance Be Used during Pregnancy? | [0] [n] |
|  |  | 8 | Improving mental health in young people | [0] [n] |
|  |  | 9 | Video directly observed therapy intervention using a mobile health application among opioid use disorder patients receiving office-based buprenorphine treatment: protocol for a pilot randomized controlled trial | [0] [n] |
|  |  | 10 | ABHD4-dependent developmental anoikis safeguards the embryonic brain | [0] [n] |
|  |  | 11 | ‘Containing the Secret of Child Sexual Abuse’.  McElvaney, Rosaleen, et al. ‘Containing the Secret of Child Sexual Abuse’. Journal of Interpersonal Violence, vol. 27, no. 6, Apr. 2012, pp. 1155–75. PubMed, https://doi.org/10.1177/0886260511424503. | [0] [n] |
|  |  | 12 | Drewett, Olivia, et al. ‘A Discussion of the Use of Virtual Reality for Training Healthcare Practitioners to Recognize Child Protection Issues’. Frontiers in Public Health, vol. 7, 2019, p. 255. PubMed, https://doi.org/10.3389/fpubh.2019.00255. | [2], [n] |
|  |  | 13 | Knowledge, Attitudes, and Perceptions Associated With Antimicrobial Stewardship Among Veterinary Students: A Multi-Country Survey From Nigeria, South Africa, and Sudan | [0] [n] |
|  |  | 14 | Kuehnle, K. ‘Child Sexual Abuse Evaluations: The Scientist-Practitioner Model’. Behavioral Sciences & the Law, vol. 16, no. 1, 1998, pp. 5–20. PubMed, https://doi.org/10.1002/(sici)1099-0798(199824)16:1<5::aid-bsl290>3.0.co;2-3. | [2,3][n] |
|  |  | 15 | Langdridge, Darren, et al. ‘A Visual Affective Analysis of Mass Media Interventions to Increase Antimicrobial Stewardship amongst the Public’. British Journal of Health Psychology, vol. 24, no. 1, Feb. 2019, pp. 66–87. PubMed, https://doi.org/10.1111/bjhp.12339. | 0, n |
|  |  | 16 | Kuchma, V. R. ‘[Healthcare of children and adolescents in national strategy for action for children for 2012-2017]’. Gigiena I Sanitariia, no. 6, Dec. 2013, pp. 26–30. | 1,3, n |
|  |  | 17 | Girinimbine from curry leaves promotes gastro protection against ethanol induced peptic ulcers and improves healing via regulation of anti-inflammatory and antioxidant mechanisms | 0, n |
|  |  | 18 | Mackey, Tim Ken, et al. ‘Health Domains for Sale: The Need for Global Health Internet Governance’. Journal of Medical Internet Research, vol. 16, no. 3, Mar. 2014, p. e62. PubMed, https://doi.org/10.2196/jmir.3276 | 0, n |
|  |  | 19 | Bond, Emma, and Katie Tyrrell. ‘Understanding Revenge Pornography: A National Survey of Police Officers and Staff in England and Wales’. Journal of Interpersonal Violence, vol. 36, no. 5–6, Mar. 2021, pp. 2166–81. PubMed, https://doi.org/10.1177/0886260518760011. | 1, 3, n |
|  |  | 20 | Firestone, Michelle, et al. ‘Fentanyl Use among Street Drug Users in Toronto, Canada: Behavioural Dynamics and Public Health Implications’. The International Journal on Drug Policy, vol. 20, no. 1, Jan. 2009, pp. 90–92. PubMed, https://doi.org/10.1016/j.drugpo.2008.02.016. | 0, n |
|  |  | 21 | Tomes, J. ‘Understanding the Referral Services Safe Harbor’. Healthcare Financial Management: Journal of the Healthcare Financial Management Association, vol. 48, no. 2, Feb. 1994, pp. 32–34, 36. | 0, n |
|  |  | 22 | Clark, Maria, et al. ‘How Public Health Nurses’ Deal with Sexting among Young People: A Qualitative Inquiry Using the Critical Incident Technique’. BMC Public Health, vol. 18, no. 1, June 2018, p. 729. PubMed, https://doi.org/10.1186/s12889-018-5642-z | 1,2,3, Y |
|  |  | 23 | Liu, Han-Hsi. ‘Use and Disclosure of Health Information and Protection of Patient Privacy in Taiwan’. Medicine and Law, vol. 29, no. 1, Mar. 2010, pp. 87–101. | 0, n |
|  |  | 24 | Blum, R. ‘Television and Teens: Health Implications. Executive Summary’. Journal of Adolescent Health Care: Official Publication of the Society for Adolescent Medicine, vol. 11, no. 1, Jan. 1990, pp. 86–90. PubMed, https://doi.org/10.1016/0197-0070(90)90135-o. | 0, n |
|  |  | 25 | Patrikas, E. O. ‘Electronic Databases and Privacy Protection: Issues for a Free Society’. Topics in Health Information Management, vol. 14, no. 1, Aug. 1993, pp. 62–68. | 0, n |
|  |  | 26 | Patrikas, E. O. ‘Electronic Databases and Privacy Protection: Issues for a Free Society’. Topics in Health Information Management, vol. 14, no. 1, Aug. 1993, pp. 62–68. | 0, n |
| **[Synonyms]** | “Safeguarding” AND “Digital” AND “Abuse” | 1 | Levine, Diane Thembekile, et al. ‘Child Safety, Protection, and Safeguarding in the Time of COVID-19 in Great Britain: Proposing a Conceptual Framework’. Child Abuse & Neglect, vol. 110, no. Pt 2, Dec. 2020, p. 104668. PubMed, https://doi.org/10.1016/j.chiabu.2020.104668. | 0, n |
|  |  | 2 | Saksena, Nivedita, et al. ‘Rebooting Consent in the Digital Age: A Governance Framework for Health Data Exchange’. BMJ Global Health, vol. 6, no. Suppl 5, July 2021, p. e005057. PubMed, https://doi.org/10.1136/bmjgh-2021-005057. | 0, n |
|  |  | 3 | Bajada, S., and A. Touraine. ‘Transcutaneous Sympathetic Stimulation: Effects of Autonomic Nervous Function’. Clinical and Experimental Neurology, vol. 17, 1981, pp. 139–45. | 0, n |
|  |  | 4 | Clark, Maria, et al. ‘How Public Health Nurses’ Deal with Sexting among Young People: A Qualitative Inquiry Using the Critical Incident Technique’. BMC Public Health, vol. 18, no. 1, June 2018, p. 729. PubMed, https://doi.org/10.1186/s12889-018-5642-z | Repeat |
|  |  |  |  |  |
| **Cochrane Library** | “Safeguarding” AND “Technology” AND “Abuse” | **0** | Zero Studies returned | 0, n |
|  | “Safeguarding” AND “Digital” AND “Abuse” | **1 Result** | NCT00176449. ‘A Comparison of Bupropion SR and Placebo for Smoking Cessation’. Https://Clinicaltrials.Gov/Show/NCT00176449, Jan. 2020. www.cochranelibrary.com, https://doi.org/10.1002/central/CN-02046330. | 0, n |
|  |  |  |  |  |
| **Scopus** | “Safeguarding” AND “**Digital**” AND “Abuse” | **17 Results** |  |  |
|  |  | 1 | Domestic abuse and intimate partner violence: the role of digital by design | [1] [n] |
|  |  | 2 | On (not) learning from self-neglect safeguarding adult reviews | [0] [n] |
|  |  | 3 | Safeguarding adult reviews: informing and enriching policy and practice on self-neglect | [0] |
|  |  | 4 | Abuse through sexual image sharing in schools: Response and responsibility | [1,3] [n] |
|  |  | 5 | Snapchat and child sexual abuse in sport: Protecting child athletes in the social media | [1, 3] [n] |
|  |  | 6 | Digital identity - Language identity - Digital communication environment \| [Digitális identitás - nyelvi identitás - digitális kommunikációs környezet] | [0] [n] |
|  |  | 7 | COVID-19 responses and human rights in selected African countries |  |
|  |  | 8 | Nonmedical prescription psychiatric drug use and the darknet: A cryptomarket analysis Open Access | [0] [n] |
|  |  | 9 | Technology-facilitated harm to individuals and society: Cases of minor’s self-produced sexual content in Russia | [1] [n] |
|  |  | 10 | Blockchain for modern digital forensics: The chain-of-custody as a distributed ledger | [0] [n] |
|  |  | 12 | Combatting those who intentionally access images depicting child sexual abuse on the Internet: A call for a new offence in England and Wales | [1, 3] [n] |
|  |  | 13 | Towards typologies of virtual maltreatment: sport, digital cultures & dark leisure | [0] [n] |
|  |  | 14 | Considerations in identifying pediatric dental neglect and the legal obligation to report | [0] [n] |
|  |  | 15 | Not All that Is Solid Melts into Air? Care-Experienced Young People, Friendship and Relationships in the 'Digital Age' | [1, 3] [n] |
|  |  | 16 | Cryptanalysis and enhancement of a distributed fine-grained access control in wireless sensor networks | [0] [n] |
|  |  | 17 | Confronting commercial sexual exploitation and sex trafficking of minors in the United States | [1, 3] [n] |
|  | “Safeguarding” AND “**Technology**” AND “Abuse” | **14 Results** |  |  |
|  |  | 1 | Blockchain and iomt against physical abuse: Bullying in schools as a case study | 3, n |
|  |  | 2 | A Discussion of the Use of Virtual Reality for Training Healthcare Practitioners to Recognize Child Protection Issues | Repeat |
|  |  | 3 | 'You don’t realize what you see!': the institutional context of emotional abuse in elite youth sport | 0, n |
|  |  | 4 | Role of ICTs in safeguarding migrant workers Thinyane H. | 0, n |
|  |  | 5 | From "intrusive" and "excessive" to financially abusive? Charitable and religious fund-raising amongst vulnerable older people | 1, n |
|  |  | 6 | Child Protection: International Issues  Hackett S. | 1, 3, n |
|  |  | 7 | Surveillance technologies in care homes: Seven principles for their use | 2, 3, n |
|  |  | 8 | Safeguarding and protecting children in the early years  Reid J.a, Burton S.b | 1, 3, n |
|  |  | 9 | Kuchma, V. R. ‘[Healthcare of children and adolescents in national strategy for action for children for 2012-2017]’. Gigiena I Sanitariia, no. 6, Dec. 2013, pp. 26–30. | Repeat  1,3, n |
|  |  | 10 | Improving mental health in young people | [0] [n] |
|  |  | 11 | eGovernance and strategic information warfare - non military approach | 0, n |
|  |  | 12 | A framework for health care information assurance policy and compliance  Cannot S, salasm F. | 0, n |
|  |  | 13 | 'From dangerousness to risk': The growing importance of screening and surveillance systems for safeguarding and promoting the well-being of children in England | 0, n |
|  |  | 14 | European competition law, the new e-communications framework, and the consumer in the information society: More than a virtual position? | 0 , n |
